# PLPP/CIN Regulates Seizure Activity by the Differential Modulation of Calsenilin Binding to GluN1 and Kv4.2 in Mice

**DOI:** 10.3389/fnmol.2017.00303

**Published:** 2017-09-25

**Authors:** Ji-Eun Kim, Hye-Won Hyun, Su-Ji Min, Duk-Shin Lee, A Ran Jeon, Min Ju Kim, Tae-Cheon Kang

**Affiliations:** Department of Anatomy and Neurobiology, Institute of Epilepsy Research, College of Medicine, Hallym University Chuncheon, South Korea

**Keywords:** cofilin, dendritic spine, DREAM, epilepsy, F-actin, KChIP3

## Abstract

Calsenilin (CSEN) binds to Kv4.2 (an A-type K^+^ channel) as well as *N*-methyl-D-aspartate receptor (NMDAR), and modulates their activities. However, the regulatory mechanisms for CSEN-binding to Kv4.2 or NMDAR remain elusive. Here, we demonstrate the novel role of pyridoxal-5′-phosphate phosphatase/chronophin (PLPP/CIN), one of the cofilin-mediated F-actin regulators, in the CSEN binding to Kv4.2 or GluN1 (an NMDAR subunit). PLPP/CIN dephosphorylated CSEN in competition with casein kinase 1, independent of cofilin dephosphorylation. As compared to wild-type mice, PLPP/CIN transgenic (PLPP/CIN^Tg^) mice showed the enhancement of Kv4.2–CSEN binding, but the reduction in CSEN–GluN1 binding. In addition, PLPP/CIN^Tg^ mice exhibited the higher intensity (severity), duration and progression of seizures, but the longer latency of seizure on-set in response to kainic acid. PLPP/CIN knockout mice reversed these phenomena. Therefore, we suggest that PLPP/CIN-mediated CSEN dephosphorylation may play an important role in the functional coupling of NMDAR and Kv4.2, which regulates the neuronal excitability.

## Introduction

Epilepsy is one of the common neurological disorders, suffering about 1% of all people, which is characterized by the periodic occurrence of seizures exhibiting abnormal synchronized neuronal discharges. A delicate balance between excitatory and inhibitory inputs in neurons and impairment of this balance lead to the uncontrolled neuronal hyperexcitability, which initiates neuronal damage and symptomatic seizure activity. Unprovoked recurrent seizures contribute to a cyclical or progressive process of worsening epilepsy and neurological deficits including learning disabilities and memory problems (Elger et al., 2004; Kim J.E. et al., 2007; Kim et al., 2008; Seeburg and Sheng, 2008; Mantegazza et al., 2010; Kim and Kang, 2011). Although the pathophysiology underlying seizure susceptibility is relevant to channelopathy, aberrant synaptic organization, impaired glial function, inflammation, and neuronal loss (Kang et al., 2006; McNamara et al., 2006; Wetherington et al., 2008; Vezzani et al., 2011), the cellular and molecular mechanisms in epilepsy remain unclear.

Pyridoxal-5′-phosphate phosphatase/chronophin (PLPP/CIN) is a serine (S) protein phosphatase, which activates cofilin-mediated F-actin depolymerization (Gohla et al., 2005; Kim et al., 2008, 2016). We have reported that PLPP/CIN expression is up-regulated in the rat hippocampus following pilocarpine-induced status epilepticus (SE), which is closely relevant to increased neuronal excitability (Kim et al., 2008). Recently, we have also reported that PLPP/CIN transgenic (PLPP/CIN^Tg^) mice show the enhancement of *N*-methyl-D-aspartate receptor (NMDAR)-mediated synaptic plasticity, but PLPP/CIN knockout (PLPP/CIN^-/-^) mice show reduced synaptic strength without the synaptic NMDAR distribution and the heterotrimerization (Kim et al., 2016). Therefore, PLPP/CIN would be a potentially important factor to regulate seizure activity via interaction with NMDAR. However, the PLPP/CIN-mediated signal pathways available to identify seizure susceptibility are less defined.

Calsenilin (CSEN) is first identified as a Ca^2+^-regulated transcriptional repressor, contains four Ca^2+^-binding EF hand domains and belongs to the neuronal calcium sensor family (Burgoyne, 2007). CSEN has multifunctional properties to block gene expression via direct binding with the downstream regulatory element sequence in target genes (Gomez-Villafuertes et al., 2005), and to act as a partner for Kv4.x subunits (Morohashi et al., 2002). Thus, CSEN is also named downstream regulatory element antagonist modulator (DREAM) or potassium channel interacting protein 3 (KChIP3). Indeed, CSEN regulates the neuronal excitability by diminishing dendritic excitability and back-propagated action potentials accompanied by Kv4.x channels (Hoffman et al., 1997; An et al., 2000; Cai et al., 2004). Furthermore, CSEN expression is markedly decreased in the hippocampi of experimental animal model and human epilepsy patient (Hong et al., 2003). Interestingly, CSEN is one of the negative modulators of NMDAR (Zhang et al., 2010). Since NMDAR and Kv4.2 reciprocally regulate each other (Kim J. et al., 2007; Jung et al., 2008) and CSEN activity is modulated by the casein kinase 1 (CK1)-mediated phosphorylation (Choi et al., 2003), it is likely that PLPP/CIN-mediated CSEN dephosphorylation would be involved in the pathophysiology of epilepsy by modulating the bindings of CSEN to NMDAR or Kv4.2 channels. Here, we demonstrate the novel role of PLPP/CIN in CSEN dephosphorylation that regulated seizure activity induced by kainic acid (KA) via the modulation of Kv4.2–CSEN or GluN1–CSEN bindings, independent of cofilin-mediated F-actin depolymerization. Therefore, we suggest that PLPP/CIN may play an important role in the functional coupling of Kv4.2 and NMDAR via CSEN, which regulates the neuronal excitability.

## Materials and Methods

### Experimental Animals and Chemicals

PLPP/CIN^Tg^ (C57BL/6J background) and PLPP/CIN^-/-^ mice (129/SvEv-C57BL/6J background) were used in the present study. The same genetic background mice were used as control animals for each group (Kim et al., 2016). Animals were provided with a commercial diet and water *ad libitum* under controlled conditions (22 ± 2°C, 55 ± 5% and a 12:12 light/dark cycle). All reagents were obtained from Sigma–Aldrich (St. Louis, MO, United States), except as noted. The antibody used in the present study is listed in Supplementary Table 1. All experimental protocols were approved by the Animal Care and Use Committee of Hallym University.

### Surgery

Under anesthesia with isoflurane (3% induction and 1.5% maintenance in a 65:35 mixture of N_2_O:O_2_), animals were positioned over a heated pad, and core temperature was maintained 37–38°C. An infusion guide cannula with osmotic pump connector (3260PGA, Plastics One, Roanoke, VA, United States) was implanted into the ventricle (2 mm depth from bregma), and connected to an osmotic pump (1003D, Alzet, Cupertino, CA, United States) containing (1) vehicle, (2) IC261 (3 μM), or (3) CKI-7 (100 μM). The pump was subcutaneously placed in the interscapular region (Kim and Kang, 2011, 2015; Kim et al., 2014). In some animals, a standard infusion guide cannula (C315GA, Plastics One, Roanoke, VA, United States) was implanted into the same site. Thereafter, the cannula was sealed with a dummy cannula. Animals were also implanted monopolar stainless steel electrode (Plastics One, Roanoke, VA, United States) into the left dorsal hippocampus (2 mm posterior; 1.25 mm lateral; 2 mm depth from bregma). Three days after surgery, freely moving animals were given KA as followed.

### Seizure Induction and EEG Recording

After baseline recording for at least 30 min, an internal infusion cannula (C315IA, Plastics One, Roanoke, VA, United States) was inserted into the lumen of the guide cannula to inject KA (0.15 μg/μl) or 4-aminopyridine (4-AP; 0.1 μg/μl) into the ventricle over a 1-min period using a microinjection pump (1 μl/min, KD Scientific, Holliston, MA, United States). Control animals received an equal volume of normal saline instead of KA or 4-AP. EEG signals were recorded with a DAM 80 differential amplifier (0.1–3000 Hz bandpass; World Precision Instruments, United States) and the data were digitized (1000 Hz) and analyzed using LabChart Pro v7 software (AD Instruments, NSW, Australia). Time of seizure onset was defined as the time point showing paroxysmal depolarizing shift, defined as lasting more than 3 s and consisting of a rhythmic discharge between 4 and 10 Hz with amplitude of at least two times higher than the baseline EEG (Kim and Kang, 2011, 2015). Total power was measured during the 2-h recording session and normalized by the baseline value obtained from each animal. Spectrograms were automatically calculated using a Hanning sliding window with 50% overlap by LabChart Pro v7. Diazepam (Valium; Hoffman la Roche, Neuilly-sur-Seine, France; 10 mg/kg, i.p.) was administered 2 h after KA injection and repeated, as needed. After recording, animals were used for anatomical or biochemical experiments.

### *In Vitro* PLPP/CIN Phosphatase Assay

Modified *in vitro* PLPP/CIN phosphatase assay using full-length recombinant human CSEN (Abcam, Cambridge, United Kingdom) and PLPP/CIN (Abcam, Cambridge, United Kingdom) was performed as described previously (Choi et al., 2003). Phosphorylation of CSEN (10 ng/μl) was performed by incubation with 200 U/μl CK1 (New England BioLabs, Ipswich, MA, United States) in 50 mM Tris–HCl, pH 7.5, 10 mM MgCl_2_, 5 mM dithiothreitol and 100 μM ATP at 30°C for 1 h. Thereafter, the sample was portioned the same volume, added PLPP/CIN (10 ng/μl) or 50 mM Tris buffer (control), and incubated at 30°C for 1 h. Crude extracts obtained from the same PLPP/CIN^-/-^ mice were used the same method without CK1 and CSEN treatment. Thereafter, the samples were used co-precipitation and western blot analysis (see below).

### Co-immunoprecipitation and Western Blot

As described previously (Kim et al., 2016), animals were quickly decapitated, and their hippocampi were dissected out in the presence of cooled artificial cerebrospinal fluid (in mM: 124 NaCl, 5 KCl, 1.25 NaH_2_PO_4_, 26 NaHCO_3_, 10 dextrose, 1.5 MgCl_2_, and 2.5 CaCl_2_). The hippocampal tissues were lysed in radioimmunoprecipitation assay buffer (RIPA: 50 mM Tris–HCl pH 8.0; 1% Nonidet P-40; 0.5% deoxycholate; 0.1% SDS, Thermo Fisher Scientific, United States) containing protease inhibitor cocktail (Roche Applied Sciences, United States), phosphatase inhibitor cocktail (PhosSTOP^®^, Roche Applied Science, United States) and 1 mM sodium orthovanadate. Protein concentrations were determined by BCA protein assay (Pierce, United States) and equal amounts of total proteins were precipitated with the appropriate primary antibodies and protein G sepharose at 4°C overnight. Beads were collected by centrifugation, eluted in 2× SDS sample buffer, and boiled at 95°C for 5 min. Next, western blotting was performed according to standard procedures. The rabbit anti-β-actin primary antibody was used as internal reference. The signals were scanned and quantified on ImageQuant LAS 4000 system (GE health, United States). The values of each sample were normalized with the corresponding amount of β-actin.

### Immunohistochemistry, FJB Staining, Golgi Impregnation, and Analysis of Spine Morphology

Under urethane anesthesia (1.5 g/kg, i.p.), animals were perfused transcardially with 4% paraformaldehyde in 0.1 M phosphate buffer (PB, pH 7.4). Brains were post-fixed in the same fixative overnight and then cryoprotected and sectioned at 30 μm with a cryostat. Free-floating coronal sections were incubated in a mixture of primary antibodies in PBS containing 0.3% Triton X-100 and 2% normal chicken serum overnight at room temperature. Sections were also incubated in a mixture of FITC- and Cy3-conjugated secondary antisera (Amersham, Piscataway, NJ, United States, 1:200) for 1 h at room temperature (Kim et al., 2014). Some tissues were used for a conventional Fluoro-Jade B (FJB) staining (Kim et al., 2014; Kim and Kang, 2015; Hyun et al., 2016). To establish the specificity of the immunostaining, a negative control test was carried out with preimmune serum instead of the primary antibody. No immunoreactivity was observed for the negative control in any structures. Golgi impregnation was performed using FD Rapid GolgiStain^TM^ kit (FD NeuroTechnologies, Inc., MD, United States), according to the manufacturer’s instructions. All images and dendritic spine morphology were analyzed using an AxioImage M2 microscope and AxioVision Rel. 4.8 software or a confocal laser scanning microscope (LSM 710, Carl Zeiss Inc., Oberkochen, Germany).

### Statistical Analysis

After evaluating the values on normality using Shapiro–Wilk *W*-test, data were analyzed by Student’s *t*-test or ANOVA followed by Newman–Keuls *post hoc* test. A *p* < 0.05 is considered to be statistically different. Quantitative data were expressed as mean ± standard error of the mean.

## Results

### PLPP/CIN Decreases Seizure Susceptibility, but Increases the Progression of Seizures and Neuronal Damage in Response to KA

**FIGURE 1:**
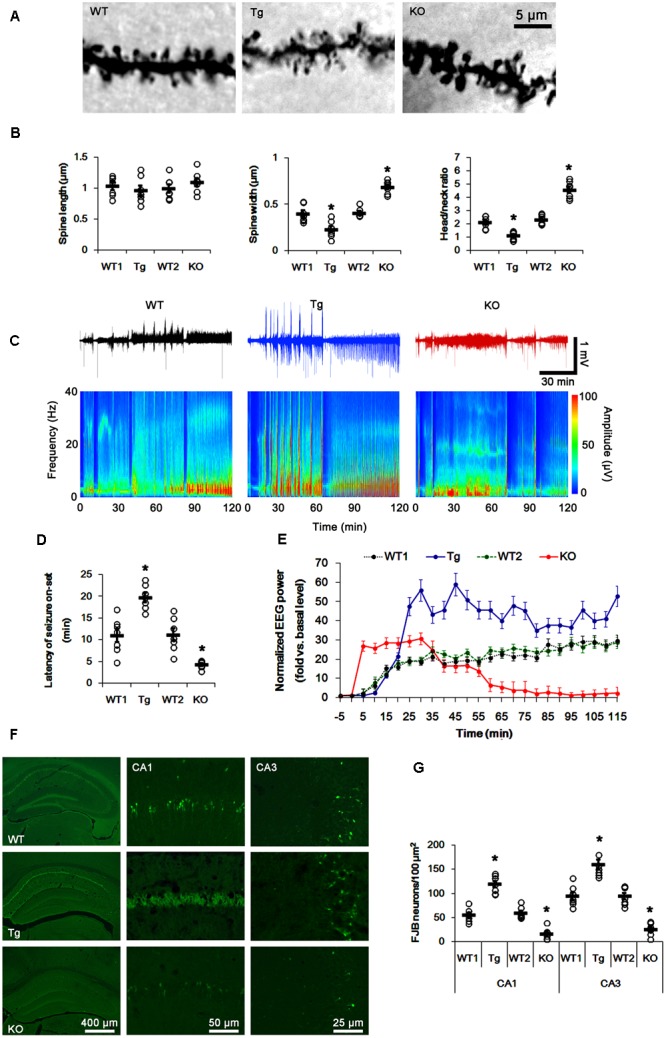
Profiles of seizure activity in PLPP/CIN^Tg^ and PLPP/CIN^-/-^ mice induce by KA. **(A,B)** Characteristics of dendritic spine morphology in PLPP/CIN^Tg^ and PLPP/CIN^-/-^ mice. As compared to WT animals, the spines in PLPP/CIN^Tg^ mice show thin necks with very small heads, while PLPP/CIN^-/-^ mice show gigantic spines. **(A)** Representative photos of dendritic spines in dentate granule cells. **(B)** Spine length, spine width, and head/neck ratio of dentate granule cells. Open circles indicate each individual value. Horizontal bars indicate mean value (mean ± SD, ^∗^*p* < 0.05 vs. WT animals; *n* = 7, respectively). **(C–E)** Seizure activity in response to KA. PLPP/CIN^Tg^ mice demonstrate increase seizure intensity, duration, and the latency of seizure on-set, as compared to PLPP/CIN^-/-^ mice. **(C)** Representative EEG traces and frequency-power spectral temporal maps in response to KA. **(D)** Quantification of latency of seizure on-set. Open circles indicate each individual value. Horizontal bars indicate mean value (mean ± SEM; ^∗^*p* < 0.05 vs. WT animals; *n* = 7, respectively). **(E)** Quantification of total EEG power (seizure intensity) in response to KA (mean ± SEM; *p* < 0.05 vs. WT animals; *n* = 7, respectively). **(F,G)** KA-induced neuronal death. One day after KA injection, neuronal death in PLPP/CIN^Tg^ mice is more severe than that in PLPP/CIN^-/-^ mice, as compared to WT animals. **(F)** Representative photos of FJB positive degenerating neurons. **(G)** Quantification of the number of FJB positive neurons in response to KA. Open circles indicate each individual value. Horizontal bars indicate mean value (mean ± SEM; ^∗^*p* < 0.05 vs. WT animals; *n* = 7, respectively).

Consistent with our previous study (Kim et al., 2016), average spine width was ∼0.40 μm and head/neck ratio was >2 in wild-type (WT, PLPP/CIN^+/+^) mice. As compared to WT animals, the spines in PLPP/CIN^Tg^ mice showed thin necks with very small heads (head/neck ratio: 1.13), while PLPP/CIN^-/-^ mice showed gigantic spines with a head/neck ratio of ∼4 (*p* < 0.05 vs. WT animals, respectively; **Figures 1A,B**). Since dendritic spines are the major integral site excitatory input, changed spine structures affect the neuronal excitability and seizure susceptibility (Wong and Guo, 2013). Thus, we measured the seizure susceptibility of PLPP/CIN^Tg^ and PLPP/CIN^-/-^ mice in response to KA.

In WT animals, the first seizure occurred 11.3 min after KA injection, progressing with time to prolonged episodes of high-frequency and high-amplitude discharges by 120 min post-KA injection (**Figures 1C–E**). EEG analyses revealed no difference in the latency of seizure on-set, duration and intensity (total EEG power) of seizures between two different genetic background WT mice. PLPP/CIN^Tg^ and PLPP/CIN^-/-^ mice showed the first seizure activity within 18.7 and 5 min after KA injection, respectively (*p* < 0.05 vs. WT animals, respectively; **Figures 1C–E**). PLPP/CIN^Tg^ mice showed increase in seizure intensity (severity) to 1.5∼3-fold of that observed in WT animal. In PLPP/CIN^-/-^ mice, seizure activity reduced ∼60 min after KA injection (*p* < 0.05 vs. WT animals, respectively; **Figures 1C–E**).

Having observed that PLPP/CIN deletion interrupted the progression of seizures in response to KA, we investigated SE-induced neuronal damage in the hippocampus 1 day post-KA injection. WT mice displayed typical CA1 and CA3 pyramidal cell loss for FJB staining (**Figures 1F,G**). As compared to WT animals, PLPP/CIN^Tg^ mice showed profound neuronal injury in the CA1 and CA3 regions, but PLPP/CIN^-/-^ mice demonstrated the overt attenuation of neuronal damage in these regions (*p* < 0.05 vs. WT animals, respectively; **Figures 1F,G**). These findings indicate that PLPP/CIN may increase seizure intensity, duration, and the latency of seizure on-set.

### F-Actin Polymerization Affects Seizure Intensity, But Not Latency of Seizure On-Set

**FIGURE 2:**
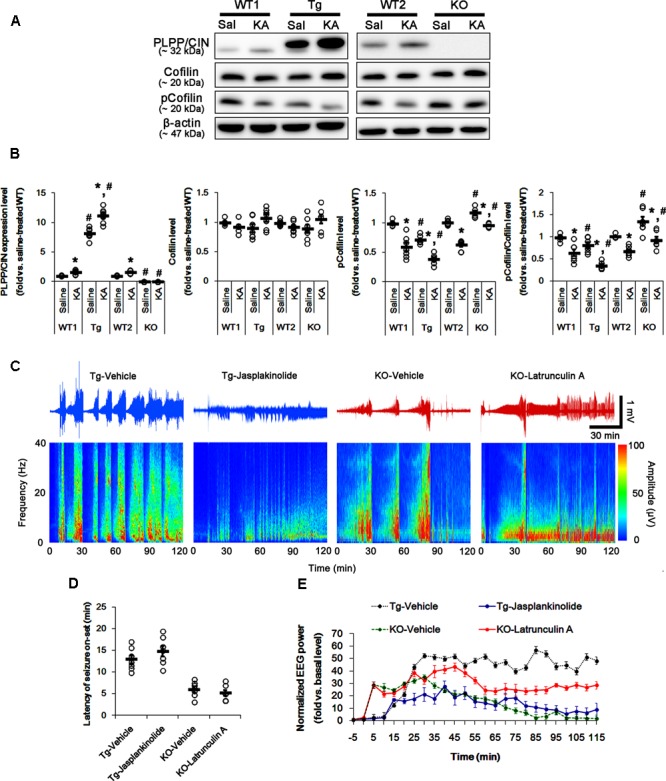
The role of F-actin dynamics in seizure activity induced by KA. **(A,B)** Changed PLPP/CIN expression and cofilin phosphorylation 2 h after KA injection. As compared to WT animals, cofilin phosphorylation is lower in PLPP/CIN^Tg^ mice, but is higher in PLPP/CIN^-/-^ mice. Western blot shows the up-regulation of PLPP/CIN expression and the reduction in pCofilin level without altered cofilin expression in WT animals. The reduced cofilin phosphorylation in PLPP/CIN^Tg^ mice is more efficient, while that in PLPP/CIN^-/-^ mice is less, as compared to WT animals. **(A)** Western blot of PLPP/CIN, cofilin, and pCofilin. **(B)** Quantification of PLPP/CIN expression, cofilin expression, and cofilin phosphorylation level based on western blot data. Open circles indicate each individual value. Horizontal bars indicate mean value (mean ± SEM; ^∗^,*^#^p* < 0.05 vs. saline-treated and WT animals, respectively; *n* = 7, respectively). **(C–E)** The effect of F-actin dynamics on seizure activity in response to KA. Jasplakinolide (an F-actin stabilizer) decreases seizure intensity (total EEG power) in PLPP/CIN^Tg^ mice, but latrunculin A (an F-actin depolymerizer) enhances it in PLPP/CIN^-/-^ animals. However, neither jasplakinolide nor latrunculin A affects the latency of seizure on-set in both groups. **(C)** Representative EEG traces and frequency-power spectral temporal maps in response to KA. **(D)** Quantification of latency of seizure on-set. Open circles indicate each individual value. Horizontal bars indicate mean value (mean ± SEM; *n* = 7, respectively). **(E)** Quantification of total EEG power (seizure intensity) in response to KA (mean ± SEM; *p* < 0.05 vs. vehicle; *n* = 7, respectively).

To elucidate the role of PLPP/CIN-mediated F-actin depolymerization in response to KA, we measured cofilin phosphorylation level 2 h after KA injection. In control group of in PLPP/CIN^Tg^ and PLPP/CIN^-/-^ mice, phospho (p)-Cofilin/Cofilin ratio were 0.8- and 1.22-fold of WT animals, respectively (*n* = 7, respectively, *p* < 0.05, respectively; **Figures 2A,B** and Supplementary Figure 3). KA increased PLPP/CIN expression, but reduced cofilin phosphorylation level in all groups except PLPP/CIN^-/-^ mice (*n* = 7, respectively, *p* < 0.05, respectively; **Figures 2A,B** and Supplementary Figure 3). The reduced cofilin phosphorylation in PLPP/CIN^Tg^ mice was more efficient than WT animals, while that in PLPP/CIN^-/-^ mice was less efficient (*n* = 7, respectively, *p* < 0.05, respectively; **Figures 2A,B** and Supplementary Figure 3). Consistent with our previous study (Kim et al., 2008), these findings indicate that PLPP/CIN may regulate cofilin phosphorylation induced by seizure activity.

Next, we explored whether F-actin depolymerization affects KA-induced seizure activity in PLPP/CIN^Tg^ and PLPP/CIN^-/-^ animals. As compared to vehicle, jasplakinolide (an F-actin stabilizer) significantly attenuated seizure intensity (total EEG power) in PLPP/CIN^Tg^ mice, while latrunculin A (an F-actin depolymerizer) increased it in PLPP/CIN^-/-^ animals (*n* = 7, respectively, *p* < 0.05 vs. vehicle, respectively; **Figures 2C–E**). However, neither jasplakinolide nor latrunculin A affected the latency of seizure on-set in both groups (**Figures 2C–E**). These findings indicate that PLPP/CIN-mediated F-actin depolymerization may contribute to the seizure intensity (severity), but not seizure threshold.

### PLPP/CIN Regulates CSEN Phosphorylation

**FIGURE 3:**
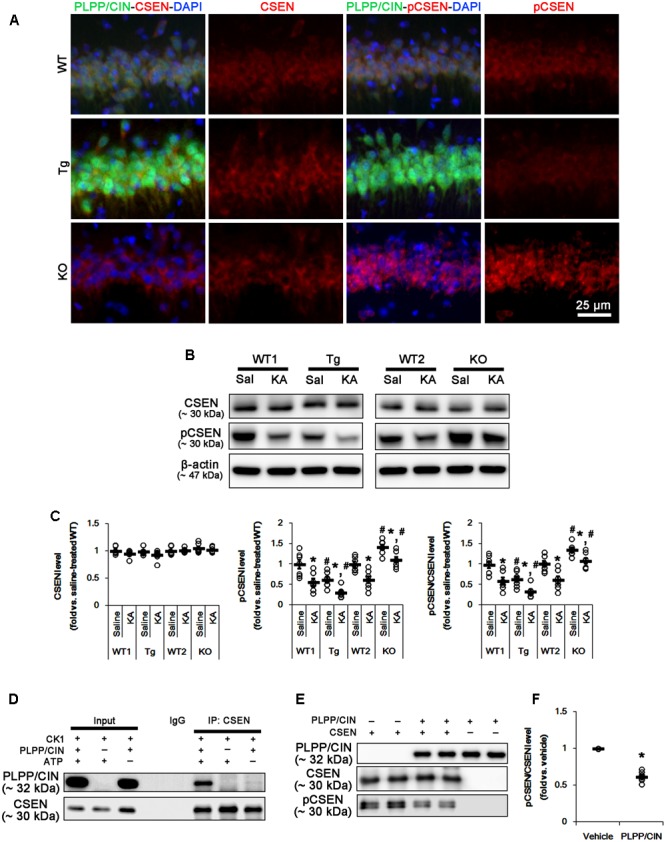
PLPP/CIN-mediated CSEN dephosphorylation. **(A)** Representative double immunofluorescent photos for PLPP/CIN and CSEN. PLPP/CIN is colocalized with CSEN in hippocampal neurons in PLPP/CIN^Tg^ mice. **(B,C)** Changed CSEN phosphorylation 2 h after KA injection. Western blot shows the CSEN phosphorylation is lower in PLPP/CIN^Tg^ mice, but is higher in PLPP/CIN^-/-^ mice. In addition, KA reduces CSEN phosphorylation without altered CSEN expression in WT animals. The reduced CSEN phosphorylation in PLPP/CIN^Tg^ mice is more efficient, while that in PLPP/CIN^-/-^ mice is less, as compared to WT animals. **(B)** Western blot of CSEN and pCSEN. **(C)** Quantification of CSEN expression and its phosphorylation level based on western blot data. Open circles indicate each individual value. Horizontal bars indicate mean value (mean ± SEM; ^∗^,^#^*p* < 0.05 vs. saline-treated and WT animals, respectively; *n* = 7, respectively). **(D–F)** *In vitro* assay using recombinant proteins. Co-immunoprecipitation and western blot reveal that PLPP/CIN binds to CSEN, and dephosphorylates CSEN (*n* = 7, *p* < 0.05 vs. vehicle). **(D)** Co-immunoprecipitation analysis of PLPP/CIN interaction with CSEN. **(E)** Western blot of PLPP/CIN, CSEN, and pCSEN. **(F)** Quantification of CSEN expression and its phosphorylation level based on western blot data. Open circles indicate each individual value. Horizontal bars indicate mean value (mean ± SEM; ^∗^*p* < 0.05 vs. vehicle; *n* = 7).

Since seizure activity affects CSEN expression in the mouse hippocampus following KA injection (Matsu-ura et al., 2002; Hong et al., 2003), we explored the effect of KA-induced seizure on CSEN expression. In control animals, double immunofluorescent study revealed that PLPP/CIN was colocalized with CSEN in hippocampal neurons, and that PLPP/CIN overexpression or deletion did not affect CSEN expression. However, PLPP/CIN overexpression decreased pCSEN level, but PLPP/CIN deletion increased it (**Figures 3A–C** and Supplementary Figure 4).

As compared to WT animals, pCSEN levels in PLPP/CIN^Tg^ and PLPP/CIN^-/-^ mice were 0.72- and 1.44-fold of WT animal levels, respectively (*n* = 7, respectively, *p* < 0.05, respectively; **Figures 3B,C** and Supplementary Figure 4). Two hours after KA injection, pCSEN level were reduced to 0.59- and 0.62-fold of control level in both WT animals without altered CSEN expression (*n* = 7, respectively, *p* < 0.05 vs. control level; **Figures 3B,C** and Supplementary Figure 4). In PLPP/CIN^Tg^ and PLPP/CIN^-/-^ mice, pCSEN levels were 0.33- and 1.07-fold of control level in WT animals, respectively (*n* = 7, respectively, *p* < 0.05 vs. control level; **Figures 3B,C** and Supplementary Figure 4).

To investigate whether other protein phosphatases affect pCSEN levels, we examined the expressions and phosphorylation levels of protein phosphatase 1A (PP1A), protein phosphatase 2A (PP2A), and protein phosphatase 2B (PP2B) after KA injection. However, KA injection did not influence on protein phosphatase expressions and phosphorylations (Supplementary Figure 1, 9). Together with increases in PLPP/CIN expression and its activity, our findings demonstrate that PLPP/CIN may dephosphorylate CSEN.

To clarify the direct phosphatase activity of PLPP/CIN on CSEN, we applied *in vitro* assay using recombinant proteins. Co-immunoprecipitation revealed that PLPP/CIN bound to CSEN, and effectively reduced CSEN phosphorylation to 62% of vehicle level (*n* = 7, *p* < 0.05 vs. vehicle; **Figures 3D–F** and Supplementary Figure 4). Taken together, our findings suggest that PLPP/CIN may directly regulate CSEN phosphorylation.

### Seizure Activity Increases the Binding of PLPP/CIN to CSEN

**FIGURE 4:**
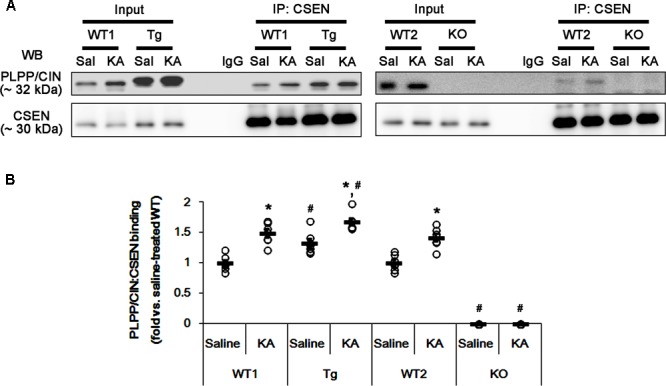
The effect of seizure activity on PLPP/CIN-mediated CSEN dephosphorylation *in vivo*. **(A,B)** Changed PLPP/CIN-CSEN bindings 2 h after KA injection. Western blot shows that PLPP/CIN-CSEN co-precipitation in PLPP/CIN^Tg^ mice is higher than WT animals under physiological condition. KA increases the binding of PLPP/CIN to CSEN in WT and PLPP/CIN^Tg^ mice. **(A)** Co-immunoprecipitation of PLPP/CIN and CSEN *in vivo*. **(B)** Co-immunoprecipitation analysis of PLPP/CIN interaction with CSEN *in vivo*. Open circles indicate each individual value. Horizontal bars indicate mean value (mean ± SEM; ^∗^,^#^*p* < 0.05 vs. saline-treated and WT animals, respectively; *n* = 7, respectively).

Next, we investigated whether seizure activity affects the binding activity of PLPP/CIN to CSEN *in vivo.* Under physiological condition (control animals), PLPP/CIN-CSEN binding in PLPP/CIN^Tg^ mice was 1.33-fold of WT animal level (*n* = 7, *p* < 0.05, respectively; **Figures 4A,B** and Supplementary Figure 5). Two hours after KA injection, the PLPP/CIN-CSEN binding in WT animals was increased to 1.5-fold of control level (*n* = 7, *p* < 0.05, **Figures 4A,B** and Supplementary Figure 5). In PLPP/CIN^Tg^ mice, the PLPP/CIN-CSEN binding was 1.7-fold of control level in WT animal (*n* = 7, *p* < 0.05, **Figures 4A,B** and Supplementary Figure 5). These findings indicate that seizure activity may increase the binding of PLPP/CIN with CSEN, which may represent activity-dependent CSEN dephosphorylation by PLPP/CIN.

### PLPP/CIN-Mediated CSEN Dephosphorylation Increases CSEN–Kv4.2 Bindings

Pharmacological blockade and genetic Kv4.2 channel deletion increase neuronal excitability and seizure susceptibility in response to KA (Magee and Carruth, 1999; Cai et al., 2004; Barnwell et al., 2009). Interestingly, CSEN plays an important role in increased functional surface expression, slower inactivation, and faster recovery from inactivation of Kv4.2 channels, which inhibit neuronal hyperactivity by generating A-type K^+^ current (*I*_A_) (Hoffman et al., 1997; An et al., 2000). Therefore, we investigated whether PLPP/CIN-mediated CSEN dephosphorylation affects the interaction of CSEN with Kv4.2, which influences on KA-induced seizures.

**FIGURE 5:**
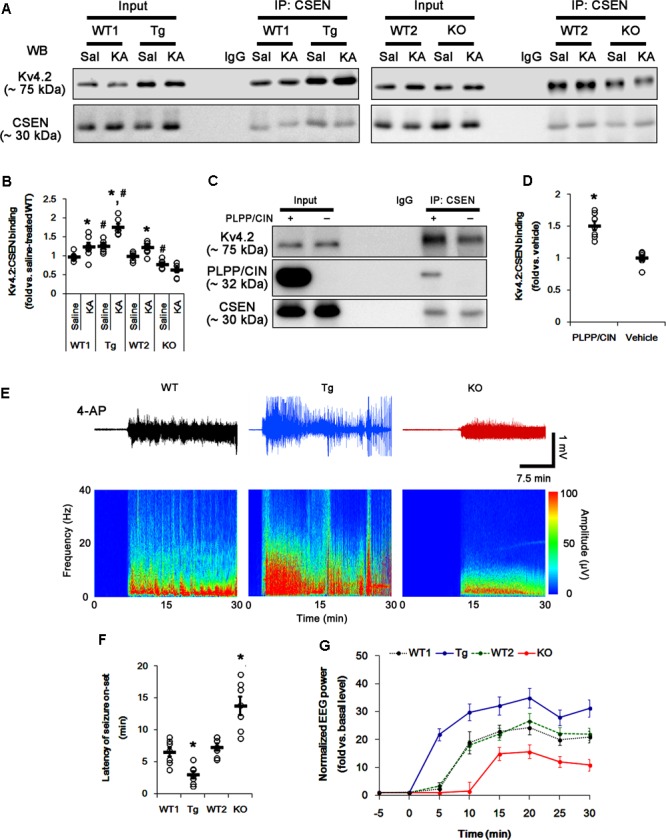
The PLPP/CIN-mediated CSEN–Kv4.2 bindings *in vivo* and *in vitro*. **(A,B)** Changed Kv4.2–CSEN bindings 2 h after KA injection. Under physiological condition, western blot shows that Kv4.2–CSEN co-precipitation is higher in PLPP/CIN^Tg^ mice, but is lower in PLPP/CIN^-/-^ mice, as compared to WT animals. KA increases the binding of CSEN to Kv4.2 in WT and PLPP/CIN^Tg^ mice. **(A)** Co-immunoprecipitation of CSEN and Kv4.2 *in vivo*. **(B)** Co-immunoprecipitation analysis of CSEN interaction with Kv4.2 *in vivo*. Open circles indicate each individual value. Horizontal bars indicate mean value (mean ± SEM; ^∗^,^#^*p* < 0.05 vs. saline-treated and WT animals, respectively; *n* = 7, respectively). **(C,D)** *In vitro* assay using crude extract obtained from PLPP/CIN^-/-^ mouse brain. PLPP/CIN treatment increases the Kv4.2–CSEN binding, as compared to vehicle. **(C)** Co-immunoprecipitation of CSEN and Kv4.2 *in vitro*. **(D)** Co-immunoprecipitation analysis of CSEN interaction with Kv4.2 *in vitro.* Open circles indicate each individual value. Horizontal bars indicate mean value (mean ± SEM; ^∗^*p* < 0.05 vs. vehicle; *n* = 7, respectively). **(E–G)** The comparison of responsiveness to 4-AP. As compared to WT animals, PLPP/CIN^Tg^ mice show the higher responsiveness to 4-AP, but PLPP/CIN^-/-^ animals reveal lower responsiveness, as compared to WT animals. **(E)** Representative EEG traces and frequency-power spectral temporal maps in response to 4-AP. **(F)** Quantification of latency of seizure on-set. Open circles indicate each individual value. Horizontal bars indicate mean value (mean ± SEM; ^∗^*p* < 0.05 vs. WT animals; *n* = 7, respectively). **(G)** Quantification of total EEG power (seizure intensity) in response to 4-AP (mean ± SEM; *p* < 0.05 vs. WT animals; *n* = 7, respectively).

In control animals, CSEN co-precipitation with Kv4.2 in PLPP/CIN^Tg^ and PLPP/CIN^-/-^ mice were 1.27- and 0.79-fold of WT animal levels (*n* = 7, respectively, *p* < 0.05, respectively; **Figures 5A,B** and Supplementary Figure 6). However, the binding of CSEN with Kv4.2 was increased by KA among all groups except PLPP/CIN^-/-^ mice (*n* = 7, respectively, *p* < 0.05; **Figures 5A,B** and Supplementary Figure 6).

To identify the role of PLPP/CIN in the binding of CSEN to Kv4.2, we applied *in vitro* assay using crude extracts from the PLPP/CIN^-/-^ brains. PLPP/CIN treatment significantly increased CSEN co-precipitation with Kv4.2 to 1.5-fold of vehicle level (*n* = 7, *p* < 0.05 vs. vehicle; **Figures 5C,D** and Supplementary Figure 6). Together with PLPP-mediated CSEN dephosphorylation (**Figure 3**), these findings suggest that the affinity of intact (dephosphorylated) CSEN to Kv4.2 may higher than that of pCSEN.

To evaluate the role of CSEN–Kv4.2 bindings in seizure activity, we analyzed the responsiveness to 4-AP (an *I*_A_ blocker; Castro et al., 2001; Cai et al., 2004) in PLPP/CIN^Tg^ and PLPP/CIN^-/-^ animals. As compared to WT animals, PLPP/CIN^Tg^ mice showed higher total EEG power (seizure intensity) and shorter latency of seizure on-set in response to 4-AP (*n* = 7, respectively, *p* < 0.05; **Figures 5E–G**). However, PLPP/CIN^-/-^ animals revealed lower responsiveness to 4-AP (*n* = 7, *p* < 0.05, respectively; **Figures 5E–G**). Since the lack of Kv4.2 channel reduces the sensitivity to 4-AP (Castro et al., 2001; Cai et al., 2004) and Kv4.2 channel plays an inhibitory role in KA-induced seizures (Barnwell et al., 2009), these findings indicate that PLPP/CIN overexpression may increase seizure threshold (the latency of seizure on-set) in response to KA through the enhanced CSEN–Kv4.2 interactions.

### PLPP/CIN-Mediated Dephosphorylation Inhibits the Binding of CSEN to GluN1 Subunit

**FIGURE 6:**
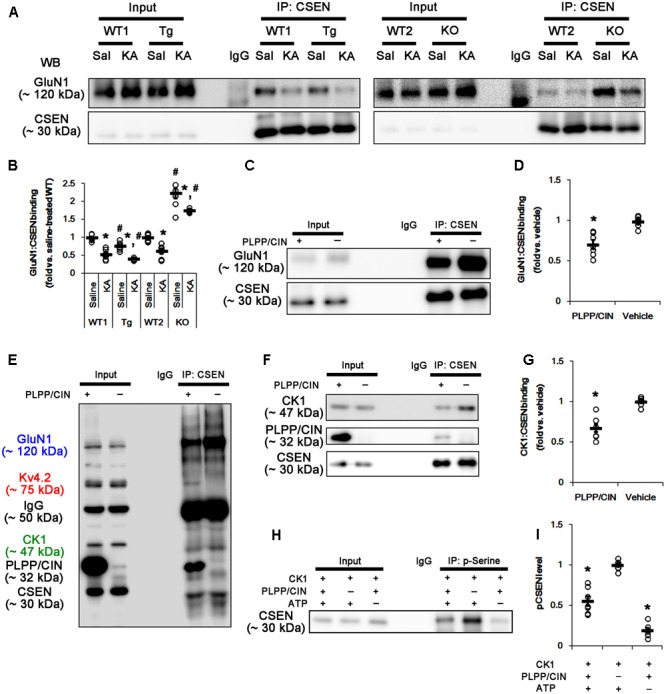
The roles of PLPP/CIN in GluN1–CSEN binding and CK1-mediated CSEN phosphorylation *in vivo* and *in vitro*. **(A,B)** Changed GluN1–CSEN bindings 2 h after KA injection. Under physiological condition, western blot shows that GluN1–CSEN co-precipitation is lower in PLPP/CIN^Tg^ mice, but is higher in PLPP/CIN^-/-^ mice, as compared to WT animals. KA decreases the binding of CSEN to GluN1 in all groups. **(A)** Co-immunoprecipitation of CSEN and GluN1 *in vivo*. **(B)** Co-immunoprecipitation analysis of CSEN interaction with GluN1 *in vivo*. Open circles indicate each individual value. Horizontal bars indicate mean value (mean ± SEM; ^∗^,^#^*p* < 0.05 vs. saline-treated and WT animals, respectively; *n* = 7, respectively). **(C,D)** *In vitro* assay using crude extract obtained from PLPP/CIN^-/-^ mouse brain. PLPP/CIN treatment decreases the GluN1–CSEN binding, as compared to vehicle. **(C)** Co-immunoprecipitation of CSEN and GluN1 *in vitro*. **(D)** Co-immunoprecipitation analysis of CSEN interaction with Kv4.2 *in vitro*. Open circles indicate each individual value. Horizontal bars indicate mean value (mean ± SEM; ^∗^*p* < 0.05 vs. vehicle; *n* = 7, respectively). **(E–I)** The competitive role of PLPP/CIN in the binding of CK1 to CSEN *in vitro*. The interactions between PLPP/CIN and CSEN are in competition with CK1. **(E)** Whole western blot image using crude extract obtained from PLPP/CIN^-/-^ mouse brain. In the presence of PLPP/CIN, the GluN1–CSEN and CK–CSEN bindings are reduced, while Kv4.2–CSEN co-precipitation is increased. **(F)** Co-immunoprecipitation of CSEN with CK1 or PLPP/CIN using crude extract obtained from PLPP/CIN^-/-^ mouse brain. **(G)** Co-immunoprecipitation analysis of CSEN interaction with CK1 or PLPP/CIN *in vitro*. Open circles indicate each individual value. Horizontal bars indicate mean value (mean ± SEM; ^∗^*p* < 0.05 vs. vehicle; *n* = 7, respectively). **(H)** *In vitro* assay using recombinant proteins. Co-immunoprecipitation reveals that PLPP/CIN inhibits CK1-mediated CSEN phosphorylation. **(I)** Co-immunoprecipitation analysis of the role of PLPP/CIN in CK1-mediated CSEN phosphorylation. Open circles indicate each individual value. Horizontal bars indicate mean value (mean ± SEM; ^∗^*p* < 0.05 vs. vehicle; *n* = 7).

Since PLPP/CIN deletion (Kim et al., 2016) and the binding of CSEN to GluN1 subunit (Zhang et al., 2010) negatively regulate NMDAR function, it is likely that PLPP/CIN-mediated CSEN dephosphorylation may reduce KA-induced seizure duration via inhibiting the CSEN-binding to GluN1 subunits. Thus, we measured the binding of CSEN with GluN1. KA injection did not affect GluN1 expression in the hippocampus of all groups (Supplementary Figures 2A,B, 10). Under physiological condition, the bindings of CSEN with GluN1 were 0.76- and 2.23-fold of WT animal level in PLPP/CIN^Tg^ and PLPP/CIN^-/-^ mice, respectively (*n* = 7, respectively, *p* < 0.05 vs. WT animals; **Figures 6A,B** and Supplementary Figure 7). Two hours after KA injection, the bindings of CSEN to GluN1 were reduced to 0.53- and 0.62-fold of control level in both WT animals, respectively (*n* = 7, respectively, *p* < 0.05; **Figures 6A,B** and Supplementary Figure 7). In PLPP/CIN^Tg^ and PLPP/CIN^-/-^ mice, the CSEN binding to GluN1 were 0.42- and 1.75-fold of control level in WT animals (*n* = 7, respectively, *p* < 0.05, respectively; **Figures 6A,B** and Supplementary Figure 7). GluN1 co-precipitation with CSEN was similarly observed in PLPP/CIN^Tg^ and PLPP/CIN^-/-^ mice (Supplementary Figures 2A,C, 10). These findings indicate that PLPP/CIN-mediated CSEN dephosphorylation may play a crucial role in the reduced binding of CSEN to GluN1.

*In vitro* assay using crude extracts of the PLPP/CIN^-/-^ brains also revealed that the binding of CSEN to GluN1 was decreased to 0.71-fold of vehicle level in the presence of PLPP/CIN (*n* = 7, respectively, *p* < 0.05; **Figures 6C,D** and Supplementary Figure 7). However, the binding of CSEN to Kv4.2 was increased (*n* = 7, respectively, *p* < 0.05; **Figures 5C,D, 6E**). Because PLPP/CIN overexpression increases NMDAR functionality without changed NMDAR distribution and the heterotrimerization (Kim et al., 2016), our findings suggest that PLPP/CIN may potentiate NMDAR functionality by inhibiting the CSEN–GluN1 binding.

### CSEN Phosphorylation Is Regulated by the Competitive Binding with PLPP/CIN and CK1

Next, we investigated the relationship between PLPP/CIN and CK1, since CK1 phosphorylates CSEN (Choi et al., 2003). *In vitro* binding assay using crude extracts from the PLPP/CIN^-/-^ brains revealed that PLPP/CIN inhibited the binding of CSEN with CK1 to 0.68-fold of vehicle level (*n* = 7, respectively, *p* < 0.05; **Figures 6E–G** and Supplementary Figure 7). *In vitro* binding assay using recombinant proteins also demonstrated that PLPP/CIN reduced CK1-mediated CSEN phosphorylation to 0.56-fold of vehicle level (*n* = 7, respectively, *p* < 0.05; **Figures 6H,I**). Taken together, these findings indicate that PLPP/CIN may be one of the counterparts of CK1 for CSEN phosphorylation.

### CSEN Phosphorylation Directly Regulates Seizure Activity in Response to KA Independent of Cofilin Phosphorylation

**FIGURE 7:**
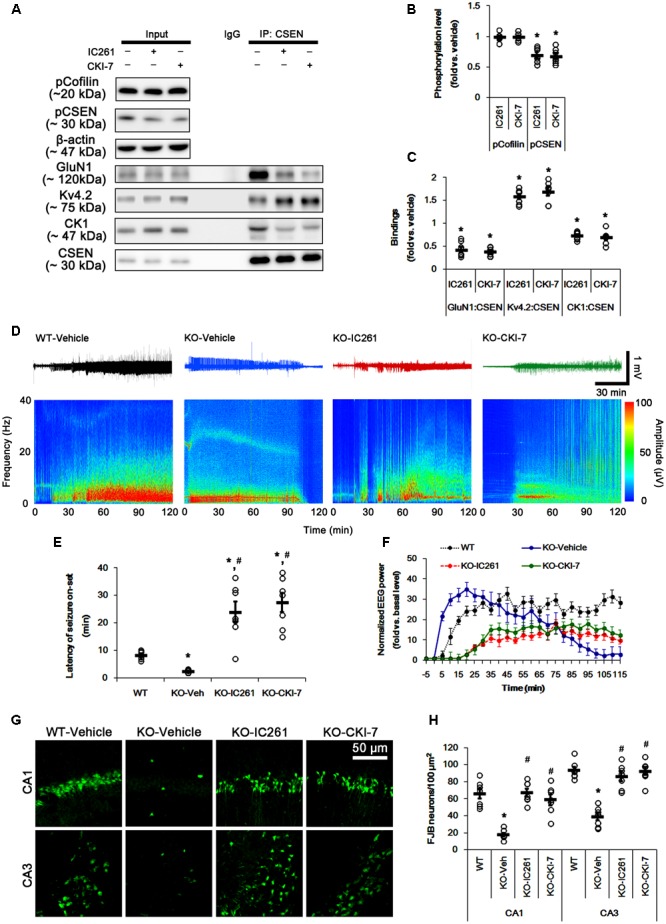
Effect of CK1 inhibitors on CSEN phosphorylation, CSEN interaction, and seizure activity in PLPP/CIN^-/-^ mice. **(A–C)** The effect of CK1 inhibitors on CSEN phosphorylation and CSEN interaction with GluN1 and Kv4.2. IC261 and CKI-7 cannot change pCofilin level. Both CK1 inhibitors decrease pCSEN level, and CK1–CSEN and GluN1–CSEN bindings. However, CK1 inhibitors increase Kv4.2–CSEN co-precipitation. **(A)** Western blot for pCSEN and pCofilin, and co-immunoprecipitation of CSEN and GluN1, Kv4.2 or CK1 *in vivo*. **(B)** Quantification of pCofilin and pCSEN levels based on western blot data. Open circles indicate each individual value. Horizontal bars indicate mean value (mean ± SEM; ^∗^*p* < 0.05 vs. vehicle; *n* = 7). **(C)** Co-immunoprecipitation analysis of CSEN interaction with GluN1, Kv4.2, and CK1 *in vivo*. Open circles indicate each individual value. Horizontal bars indicate mean value (mean ± SEM; ^∗^*p* < 0.05 vs. vehicle; *n* = 7, respectively). **(D–F)** The effect of CK1 inhibitors on seizure activity in response to KA. CK1 inhibitors increase the latency of seizure on-set and the seizure duration in response to KA. **(D)** Representative EEG traces and frequency-power spectral temporal maps in response to KA. **(E)** Quantification of latency of seizure on-set. Open circles indicate each individual value. Horizontal bars indicate mean value (mean ± SEM; ^∗^,^#^*p* < 0.05 vs. WT animals and vehicle, respectively; *n* = 7, respectively). **(F)** Quantification of total EEG power (seizure intensity) in response to KA (mean ± SEM; *p* < 0.05 vs. WT animals and vehicle; *n* = 7, respectively). **(G,H)** Effect of CK1 inhibitors on KA-induced neuronal death. One day after KA injection, both CK1 inhibitors exacerbate neuronal death, as compared to vehicle. **(G)** Representative photos of FJB positive degenerating neurons. **(H)** Quantification of the number of FJB positive neurons in response to KA. Open circles indicate each individual value. Horizontal bars indicate mean value (mean ± SEM; ^∗^,^#^*p* < 0.05 vs. WT animals and vehicle, respectively; *n* = 7, respectively).

The remaining question is whether CSEN phosphorylation affects seizure activity in response to KA by regulating GluN1–CSEN and Kv4.2–CSEN binding *in vivo*. Thus, we applied two CK1 inhibitors (IC261 and CKI-7) in PLPP/CIN^-/-^ mice to decrease CSEN phosphorylation. Neither IC261 nor CKI-7 affected pCofilin level. Both CK1 inhibitors inhibited the binding of CK1 to CSEN, and reduced pCSEN level as well as GluN1–CSEN bindings (*n* = 7, respectively, *p* < 0.05; **Figures 7A–C** and Supplementary Figure 8), However, CK1 inhibitions increased Kv4.2–CSEN co-precipitation (*n* = 7, respectively, *p* < 0.05; **Figures 7A–C** and Supplementary Figure 8). In addition, CK1 inhibitors increased the latency of seizure on-set, the seizure duration and seizure-induced neuronal damage in response to KA (*n* = 7, respectively, *p* < 0.05 vs. vehicle; **Figures 7D–H**). These findings indicate that CSEN may directly regulate the seizure susceptibility and its progression by differential modulation of its interaction with Kv4.2 and GluN1 subunit.

## Discussion

The present study investigated the involvement of PLPP/CIN in the seizure activity in response to KA in mice. This study demonstrated four major findings: (1) PLPP/CIN-mediated F-actin depolymerization correlated with the seizure intensity (total EEG power) in response to KA. (2) PLPP/CIN dephosphorylated CSEN, reflecting that CSEN is the novel substrate for PLPP/CIN. (3) PLPP/CIN-mediated CSEN dephosphorylation facilitated the Kv4.2–CSEN bindings, which increased seizure threshold (the latency of seizure on-set) in response to KA. (4) PLPP/CIN-mediated CSEN dephosphorylation inhibited the binding of CSEN to GluN1, which prolonged the seizure duration.

PLPP/CIN is firstly discovered as a phosphatase for pyridoxal-5′-phosphate (PLP, an active form of vitamin B_6_; Turner and Happold, 1961), and later identified as a serine specific phosphatase of the non-thiol-based haloacid dehalogenase superfamily of hydrolases and a modulator for cofilin activity (Gohla et al., 2005; Kim et al., 2008, 2016). In our previous study (Kim et al., 2008), Seizure activity elevates PLPP/CIN expression in the rat hippocampus accompanied by increase in neuronal excitability. In the present study, the genetic PLPP/CIN manipulation changed seizure activity in response to KA. PLP plays a role as a cofactor in γ-aminobutyric acid (GABA) synthesis by glutamate decarboxylase (Miller et al., 1978). Thus, genetic defect of PLP metabolism results in vitamin B_6_-dependent epilepsy, which is an autosomal recessive epileptic encephalopathy responsive to pharmacological dosages of pyridoxine (a PLP precursor) and resistant to antiepileptic drugs (Stockler et al., 2011). With respect to these reports, altered seizure activity in PLPP/CIN^Tg^ and PLPP/CIN^-/-^ mice would be affected by PLPP/CIN-mediated alterations in GABAergic inhibitory neurotransmission via changed PLP concentration. However, this hypothesis can be excluded because the PLP concentration in the brain is tightly regulated by two rate-limiting synthetic enzymes, pyridoxal kinase and pyridoxine-5′-phosphate oxidase (Choi et al., 1983; Ubbink et al., 1990). Indeed, pyridoxine could not inhibit seizure activity in rats (Kim et al., 2008). Furthermore, both PLPP/CIN^Tg^ and PLPP/CIN^-/-^ mice show the similar GABAergic transmission under physiological condition (Kim et al., 2016).

In the present study, we found that F-actin polymerization by jasplakinolide significantly attenuated seizure intensity in PLPP/CIN^Tg^ mice, but F-actin depolymerization by latrunculin A increased it in PLPP/CIN^-/-^ animals. These findings indicate that PLPP/CIN-mediated F-actin depolymerization itself affects KA-induced seizure intensity. However, neither jasplakinolide nor latrunculin A affected the latency of seizure on-set in both groups. Thus, it is likely that the unknown PLPP/CIN-mediated mechanism may underlie changed seizure susceptibility and its duration. In the present study, PLPP/CIN bound to CSEN and dephosphorylated it *in vivo* and *in vitro*. In addition, these interactions between PLPP/CIN and CSEN were in competition with CK1 that phosphorylated CSEN. These findings are the first evidence providing the role of PLPP/CIN as a CSEN dephosphorylating enzyme. Furthermore, PLPP/CIN-mediated CSEN dephosphorylation inhibited the GluN1–CSEN binding, but enhanced the Kv4.2–CSEN binding *in vivo* and *in vitro*. These findings also provide the novel biological functions of PLPP/CIN-mediated CSEN dephosphorylation.

CSEN modulates multiple intracellular events related to pain (Cheng et al., 2002), long-term potentiation (An et al., 2000), learning and memory (Lilliehook et al., 2003; Alexander et al., 2009). In particular, CSEN plays an important role in maintenance of Kv4.2 channel functionality (Hoffman et al., 1997; An et al., 2000). Kv4.2 channels are pore-forming subunits of the neuronal somatodendritic *I*_A_, which inhibits neuronal hyperexcitability by dampening dendritic excitability and back-propagated action potentials (Hoffman et al., 1997; Cai et al., 2004). CSEN binds to Kv4.2 channels and promotes Kv4.2 channel functionality (An et al., 2000). Indeed, CSEN deletion reduces *I*_A_ density in hippocampal dentate granule cells in mice (Lilliehook et al., 2003). Furthermore, reductions in Kv4.2 channel expression and a mutation in the gene encoding Kv4.2 (*KCND2*) are reported in animal models of epilepsy (Tsaur et al., 1992; Monaghan et al., 2008) and patients with temporal lobe epilepsy (Singh et al., 2006). With respect to these previous reports, it is likely that PLPP/CIN-mediated changes in seizure susceptibility would be relevant to the role of CSEN as an auxiliary subunit for Kv4.2 channels, although we did not investigate the Kv4.2 functionality by a direct electrophysiological approach. Indeed, the present study demonstrates that PLPP/CIN^Tg^ mice showed significantly increases in CSEN co-precipitation with Kv4.2 and responsiveness to 4-AP. In addition, PLPP/CIN^-/-^ mice reversed to these phenomena. Therefore, our findings provide the possibility that PLPP/CIN may decrease seizure susceptibility via the enhancing CSEN–Kv4.2 interactions.

**FIGURE 8:**
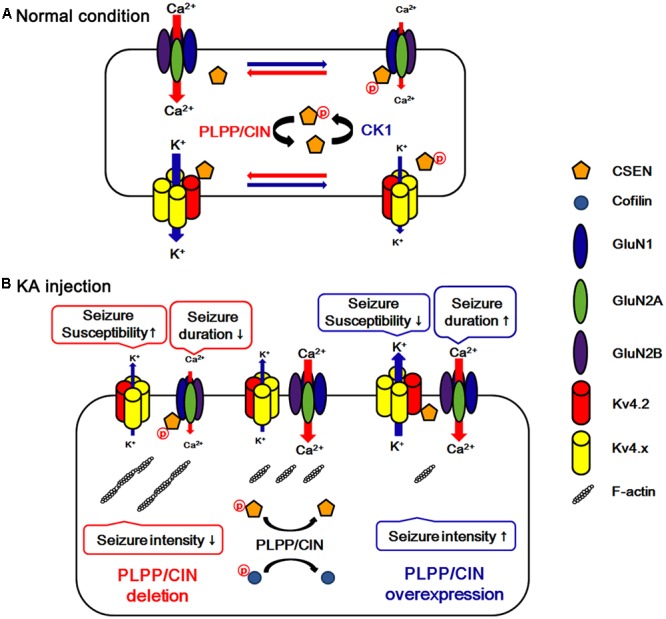
Hypothesized role of PLPP/CIN -mediated CSEN regulation in normal condition **(A)** and seizure susceptibility in response to KA injection **(B)**. PLPP/CIN dephosphorylates CSEN, which has the distinct affinity to bind with Kv4.2 and GluN1, independent of cofilin-mediated F-actin depolymerization. Dephosphorylated CSEN binds to Kv4.2, which facilitates Kv4.2 activity. However, phosphorylated CSEN binds to GluN1, and inhibits NMDAR activity. These PLPP/CIN-mediated functional coupling of NMDAR and Kv4.2 differently affects seizure susceptibility and its duration, accompanied by F-actin depolymerization.

A local increase in dendritic excitability favors the back-propagated action potentials with a subsequent boost in the NMDAR-mediated Ca^2+^ influx (Frick et al., 2004). Indeed, Kv4.2 regulates Ca^2+^ signaling through spontaneous NMDAR activation to control synaptic NMDAR expression and plasticity (Jung et al., 2008), which in turn leads to activity-dependent Kv4.2 internalization (inactivation) (Kim J. et al., 2007). Thus, it is likely that Kv4.2 and NMDAR may reciprocally regulate each other under normal condition. In the present study, we found that CSEN co-precipitation with GluN1 was decreased in PLPP/CIN^Tg^ mice under normal condition. Furthermore, PLPP/CIN^Tg^ mice showed the prolonged seizure duration and the severe progression of seizure activity in response to KA, accompanied by the rapid dissociation of CSEN from GluN1. In contrast, PLPP/CIN^-/-^ mice exhibited the spontaneous abrogation of KA-induced seizure activity with less reduced CSEN co-precipitation with GluN1. Furthermore, inhibition of CSEN phosphorylation by CK1 inhibitors delayed the latency of seizure on-set in response to KA, accompanied by reduced CSEN–GluN1 binding. Since CSEN (Zhang et al., 2010) and PLPP/CIN deletion (Kim et al., 2016) inhibit NMDAR functionality, our findings suggest that PLPP/CIN may increase KA-induced seizure duration and neuronal death due to the NMDAR activation by the reduction of the CSEN–GluN1 binding. In light of the functional coupling of NMDAR and Kv4.2 (Frick et al., 2004; Kim J. et al., 2007; Jung et al., 2008), the present study clearly demonstrates that PLPP/CIN-mediated CSEN regulation may maintain appropriate neuronal activity under physiological condition (**Figure 8A**) and have a key role in seizure susceptibility, duration, and progression (**Figure 8B**).

PLPP/CIN-mediated F-actin dynamics play an important role in the maturation of dendritic spines (Kim et al., 2016). Dendritic spines are critical for synaptic transmission, representing the primary location of excitatory synaptic inputs to neurons (Kasai et al., 2010). Thus, dendritic spine pathology (loss of dendritic spines and alterations in spine shape/size) seems to be an important pathophysiological mechanism in epilepsy (Ferhat et al., 2003; Wong, 2005). Indeed, a significant decrease in dendritic spine density is frequently seen in hippocampal pyramidal neurons and dentate granule cells in patients with temporal lobe epilepsy (Freiman et al., 2011) and epilepsy animal models (González-Burgos et al., 2004; Ampuero et al., 2007). However, the ictogenic role of dendritic spine morphology has been still controversial. For example, enhanced LIM kinase 1 (LIMK1, one of counterparts of PLPP/CIN for cofilin phosphorylation) protein translation reduces the seizure susceptibility accompanied by dendritic spine loss as well as paradoxical increased spine volume (Jimenez-Mateos et al., 2012, 2015). In the present study, PLPP/CIN^Tg^ mice were less susceptible to KA-induced seizures with small and immature dendritic spines. In contrast, PLPP/CIN^-/-^ mice were more sensitive with abnormal gigantic spines. Furthermore, jasplakinolide abolished seizure intensity (not seizure susceptibility or its duration) in PLPP/CIN^Tg^ mice, and latrunculin A enhanced it in PLPP/CIN^-/-^ animals. Since small spines are less efficient in the compartmentalization of Ca^2+^ from the dendritic shaft than large spines (Majewska et al., 2000), the present data provide the possibility that the small spine size or F-actin depolymerization may enhance seizure intensity due to increased Ca^2+^ flux from spines to the dendritic shaft. Further studies are needed to elucidate the correlation between ictogenesis and spine pathology.

## Conclusion

We found that PLPP/CIN-mediated F-actin depolymerization increased seizure intensity in response to KA. In addition, PLPP/CIN dephosphorylated CSEN, which differently affected seizure susceptibility and its duration by regulating the binding of CSEN to Kv4.2 and NMDAR. Thus, we suggest that PLPP/CIN may play an important role in the regulation of neuronal excitability via modulating CSEN phosphorylation under physiological and pathological conditions (**Figure 8**).

## Author Contributions

T-CK designed and supervised the project. J-EK performed the experiments described in the manuscript with H-WH, S-JM, D-SL, AJ, and MK. J-EK and T-CK analyzed the data and wrote the manuscript.

## Conflict of Interest Statement

The authors declare that the research was conducted in the absence of any commercial or financial relationships that could be construed as a potential conflict of interest.
