# Supplementary material for: PLPP/CIN Regulates Seizure Activity by the Differential Modulation of Calsenilin Binding to GluN1 and Kv4.2 in Mice
Source: Front Mol Neurosci. 2017 Sep 25;10:303. doi: 10.3389/fnmol.2017.00303 (PMC5622162; doi:10.3389/fnmol.2017.00303)
Supplement: Supplementary file 1 [file Data_Sheet_1.PDF]

Supplementary information

PLPP/CIN regulates seizure activity by the differential modulation of calsenilin binding to GluN1 and Kv4.2 in mice

Ji-Eun Kim, Hye-Won Hyun, Su-Ji Min, Duk-Shin Lee, A Ran Jeon, Min Ju Kim, Tae-Cheon Kang*

Department of Anatomy and Neurobiology, Institute of Epilepsy Research, College of Medicine, Hallym University, Chuncheon 200-702, South Korea.

* Correspondence should be addressed to T-C K (e-mail: tckang@hallym.ac.kr)

Supplementary Table 1. Primary antibodies used in the present study

Antigen	Host	Manufacturer (catalog number)	Dilution used
CK1δ	Mouse	Abcam (ab85320)	1:5,000 (WB)
Cofilin	Rabbit	Sigma (C8736)	1:20,000 (WB)
CSEN	Mouse<br>Rabbit	Millipore (05-756)<br>Proteintech (1203-1-AP)	1:50 (IP)<br>1:200 (IHC)/1:1,000 (WB)
GluN1	Rabbit<br>Mouse	Thermo Scientific (PA3-102)<br>Abbiotec (253182)	1:600 (WB)<br>1:250 (IP)
Kv4.2	Rabbit	Millipore (AB5360)	1:500 (WB)
p-Serine	Rabbit	Abcam (ab9332)	1:50 (IP)
pCofilin	Rabbit	Abcam (ab47281)	1:1,000 (WB)
pCSEN	Rabbit<br>Rabbit	Bioss (bs-12167R)<br>Thermo Scientific (PA5-38564)	1:500 (WB)<br>1:100 (IHC)/1:1,000 (WB)
PLPP/CIN	Rabbit<br>Mouse	Sigma (HPA001099)<br>Santa Cruz (sc-398850)	1:1,000 (WB)<br>1:200 (IHC)
PP1A	Rabbit	Abcam (ab52619)	1:5,000 (WB)
PP2A	Rabbit	Cell Signaling (#2038)	1:5,000 (WB)
PP2B	Rabbit	Millipore (07-068-I)	1:1,000 (WB)
pPP1A	Rabbit	Abcam (ab62334)	1:5,000 (WB)
pPP2A	Rabbit	Abcam (ab32104)	1:1,000 (WB)
pPP2B	Rabbit	Badrilla (A010-80)	1:1,000 (WB)
β-actin	Mouse	Sigma (A5316)	1:5,000 (WB)

IHC, Immunohistochemistry; IP, Immunoprecipitation; WB, Western blot.

Supplementary Figures

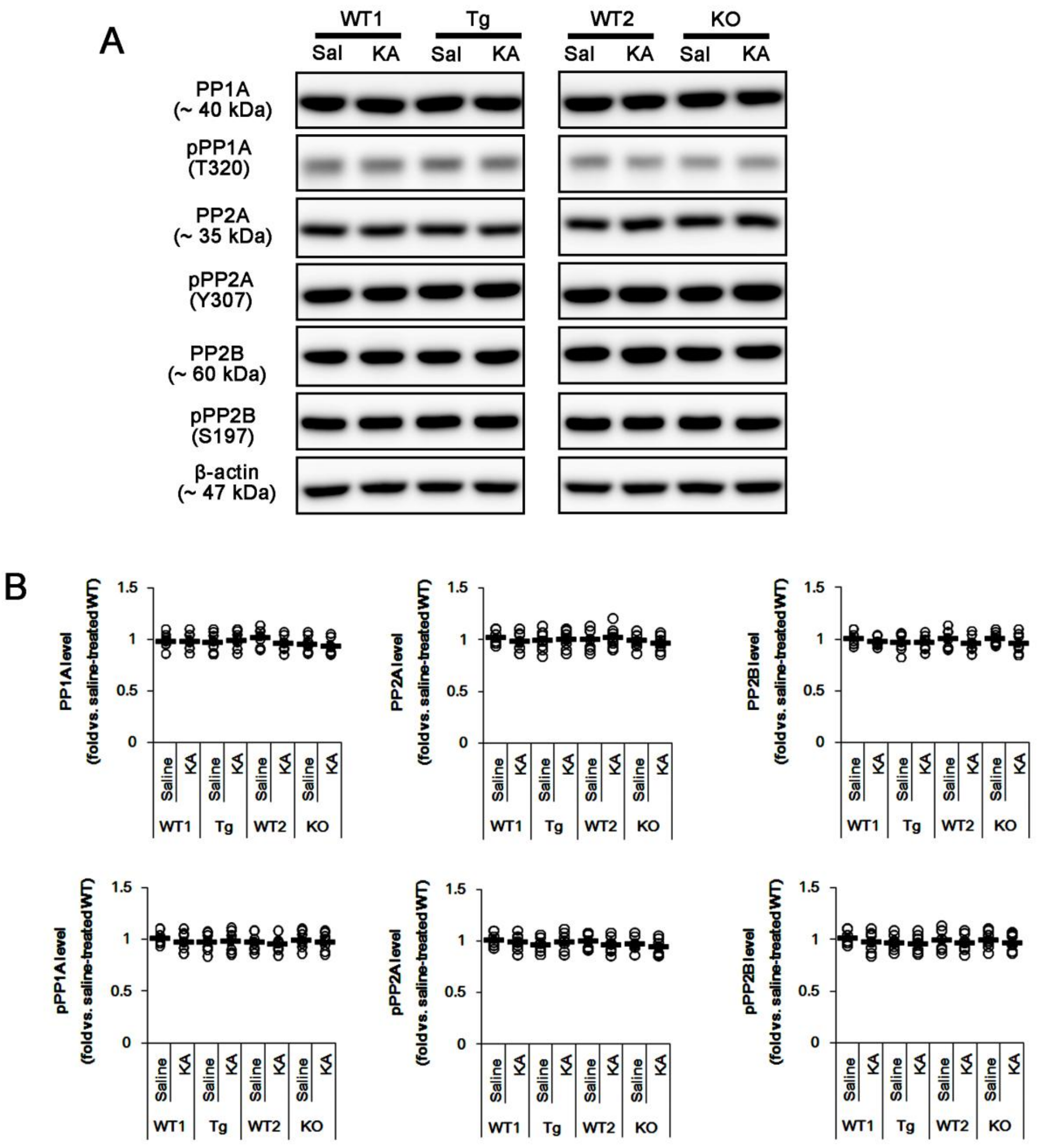

Supplementary Figure 1. : Profiles of protein phosphatases in PLPP/CIN^{Tg} and PLPP/CIN$^{-/-}$ mice induce by KA. KA injection does not affect expression/phosphorylation levels of protein phosphatases in all groups. (A) Western blot images of PP1A, PP2A, PP2B, pPP1A, pPP2A and pPP2B. (B) Quantification of PP1A, PP2A, PP2B, pPP1A, pPP2A and pPP2B level based on western blot data. Open circles indicate each individual value. Horizontal bars indicate mean value (mean ± S.E.M.; n = 7, respectively).

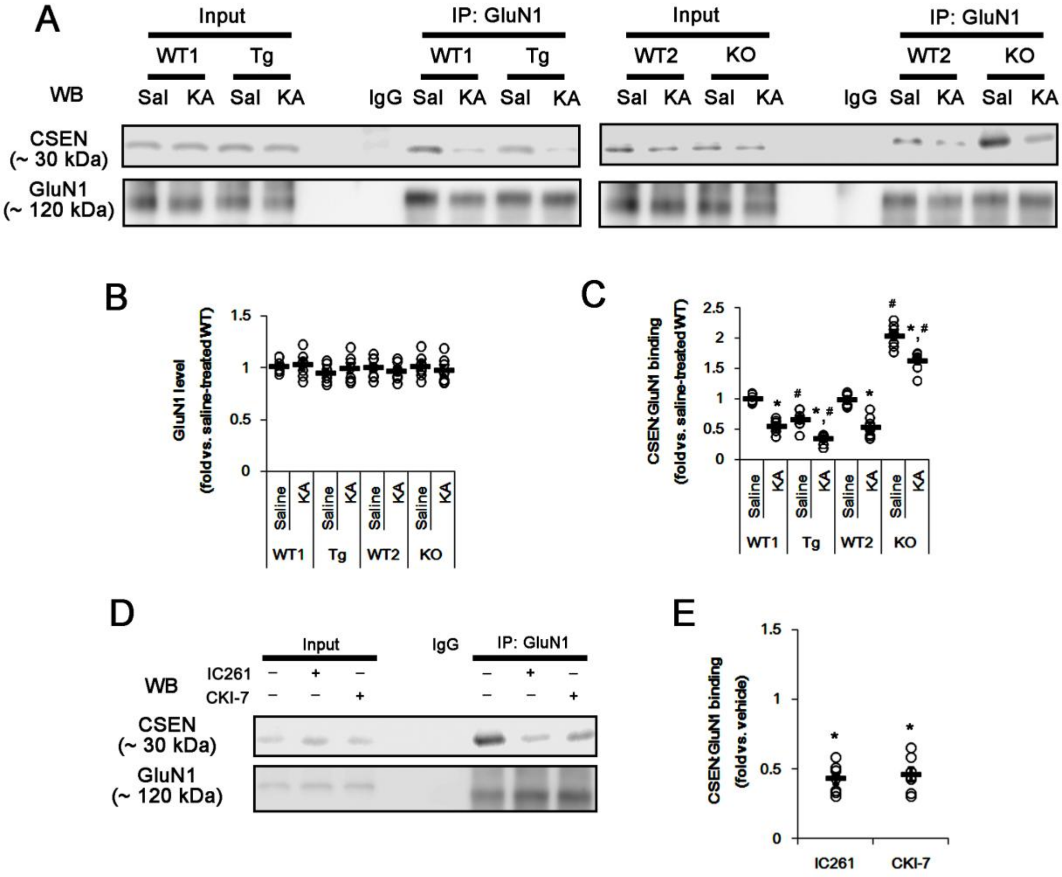

Supplementary Figure 2. : The roles of PLPP/CIN in CSEN-GluN1 binding and CK1-mediated CSEN phosphorylation *in vivo* and *in vitro*. **(A-C) Changed CSEN-GluN1 bindings 2 h after KA injection.** KA injection cannot affect GluR1 expression in all groups. Under physiological condition, CSEN-GluN1 co-precipitation is lower in PLPP/CIN^{Tg} mice, but is higher in PLPP/CIN$^{-/-}$ mice, as compared to WT animals. KA decreases the binding of CSEN to GluN1 in all groups. (A) Co-immunoprecipitation of GluN1 and CSEN *in vivo*. (B) Quantitative analysis of GluN1 expression in the hippocampus following KA injection. Open circles indicate each individual value. Horizontal bars indicate mean value (mean ± S.E.M.; n = 7, respectively). (C) Co-immunoprecipitation analysis of GluN1 interaction with CSEN *in vivo*. Open circles indicate each individual value. Horizontal bars indicate mean value (mean ± S.E.M.; $*,^{\#}p < 0.05$ vs. saline-treated and WT animals, respectively; n = 7, respectively). **(D-E) The effect of CK1 inhibitors on GluN1 interaction with CSEN.** CK1 inhibitors increase CSEN-GluN1 co-precipitation. (D) Western blot for co-immunoprecipitation of CSEN and GluN1 *in vivo*. (E) Co-immunoprecipitation analysis of GluN1 interaction with CSEN *in vivo*. Open circles indicate each individual value. Horizontal bars indicate mean value (mean ± S.E.M.; $*p < 0.05$ vs. vehicle; n = 7, respectively).

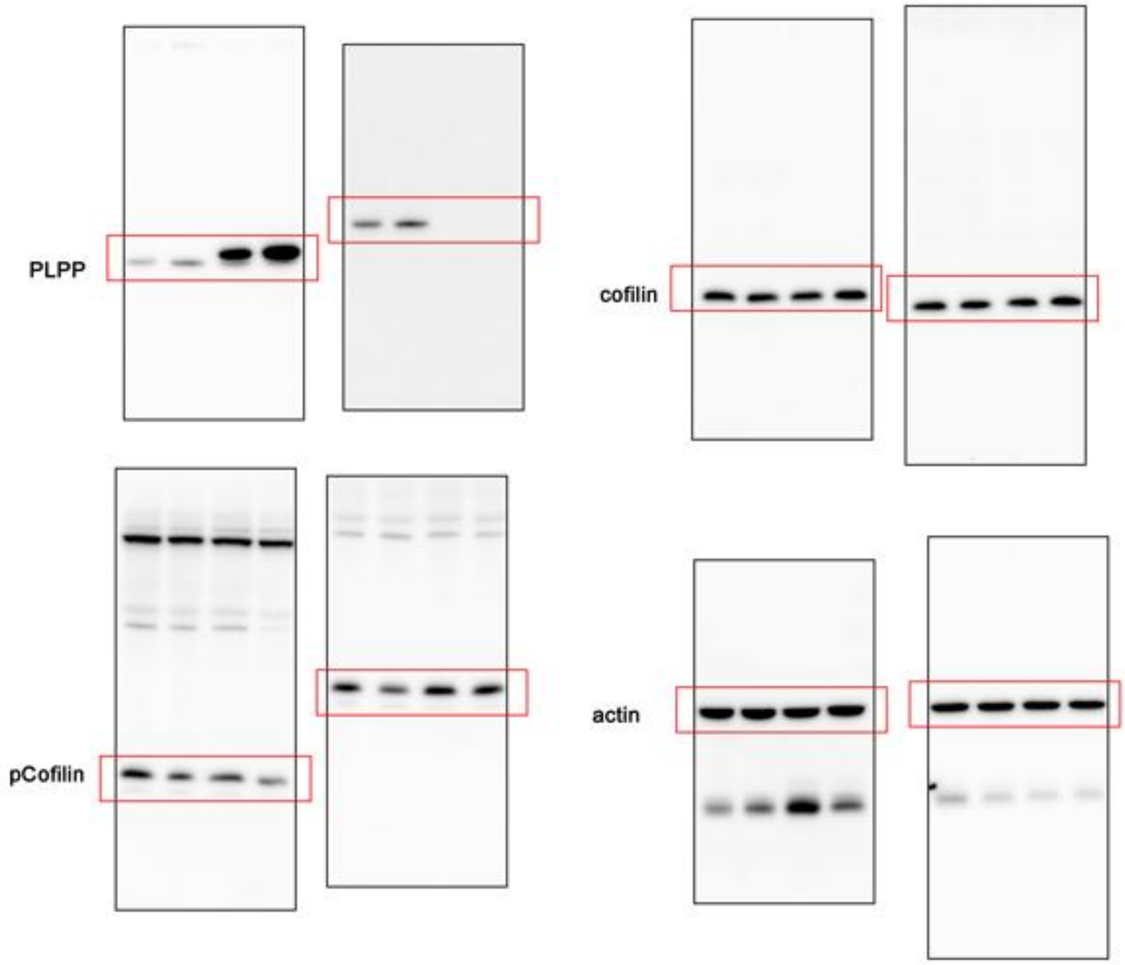

Supplementary Figure 3. : Full-length gel images of western blot data in Fig. 2. The cropped parts of western blots are indicated with boxes.

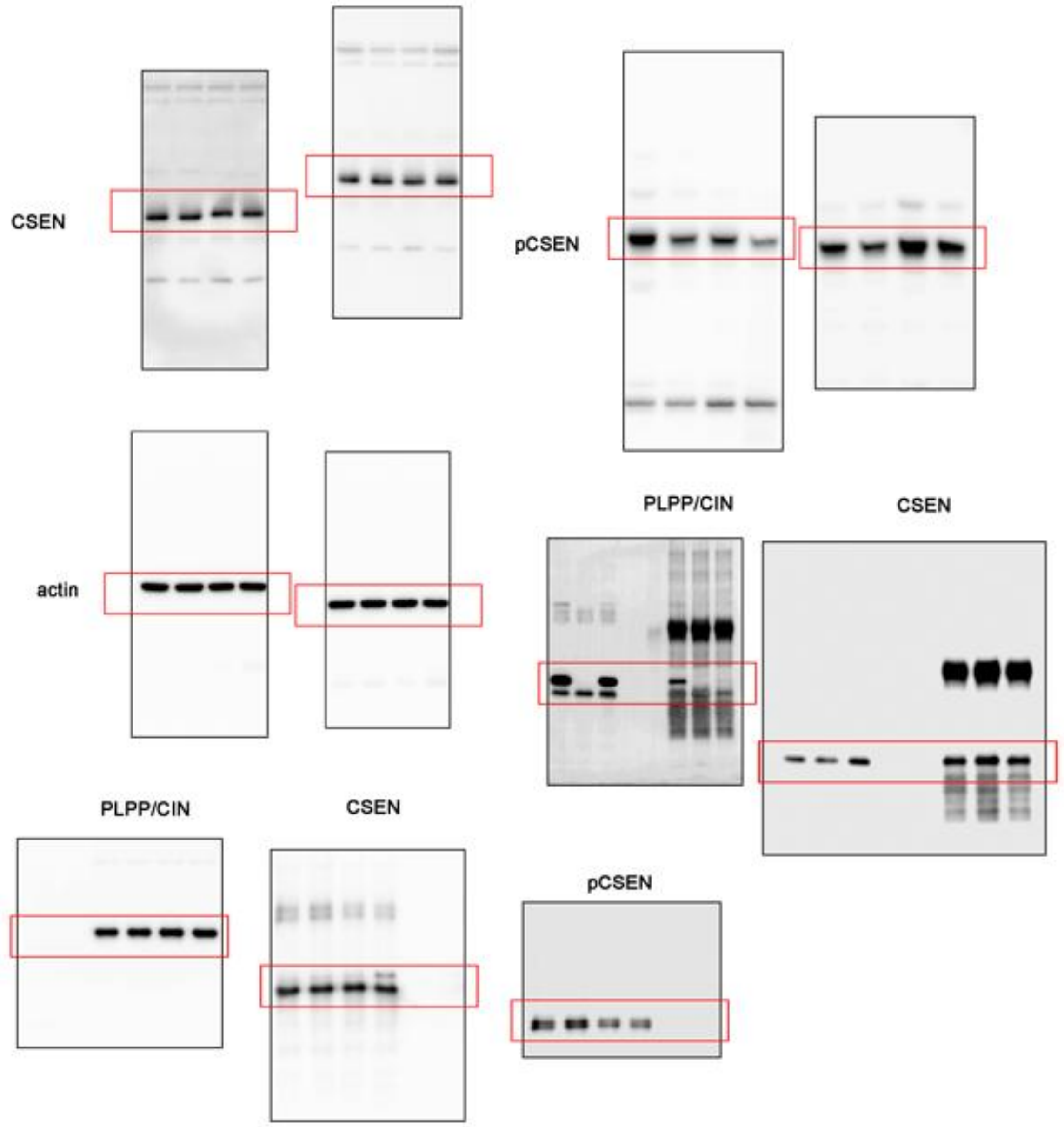

Supplementary Figure 4. : Full-length gel images of western blot data in Fig. 3. The cropped parts of western blots are indicated with boxes.

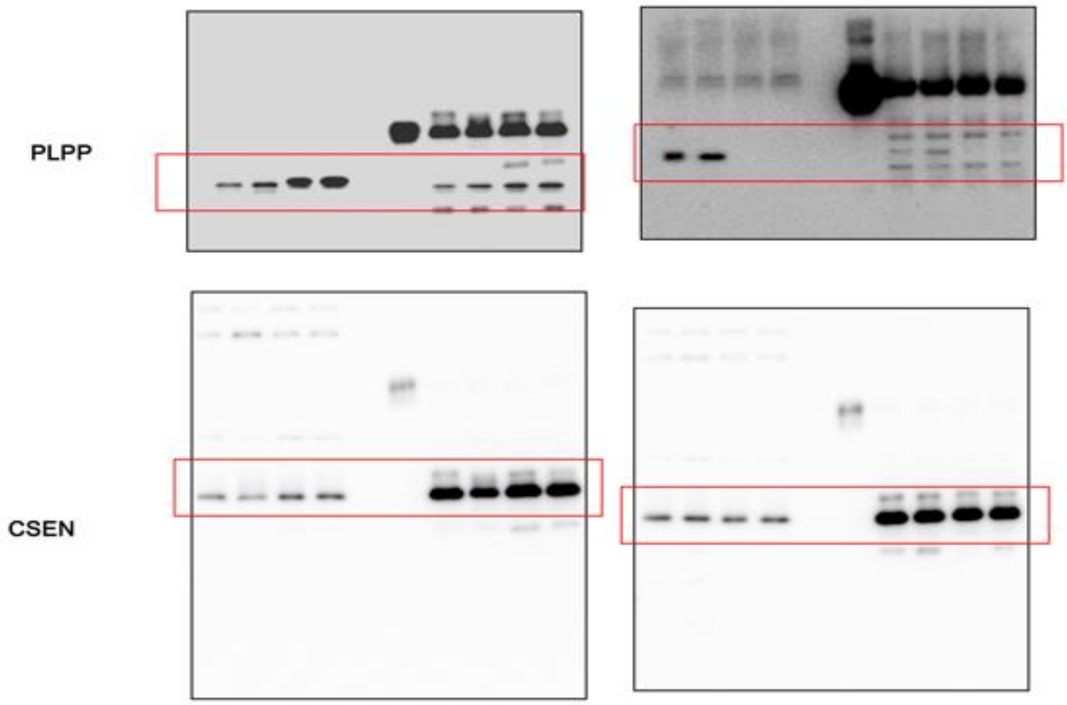

Supplementary Figure 5. : Full-length gel images of western blot data in Fig. 4. The cropped parts of western blots are indicated with boxes.

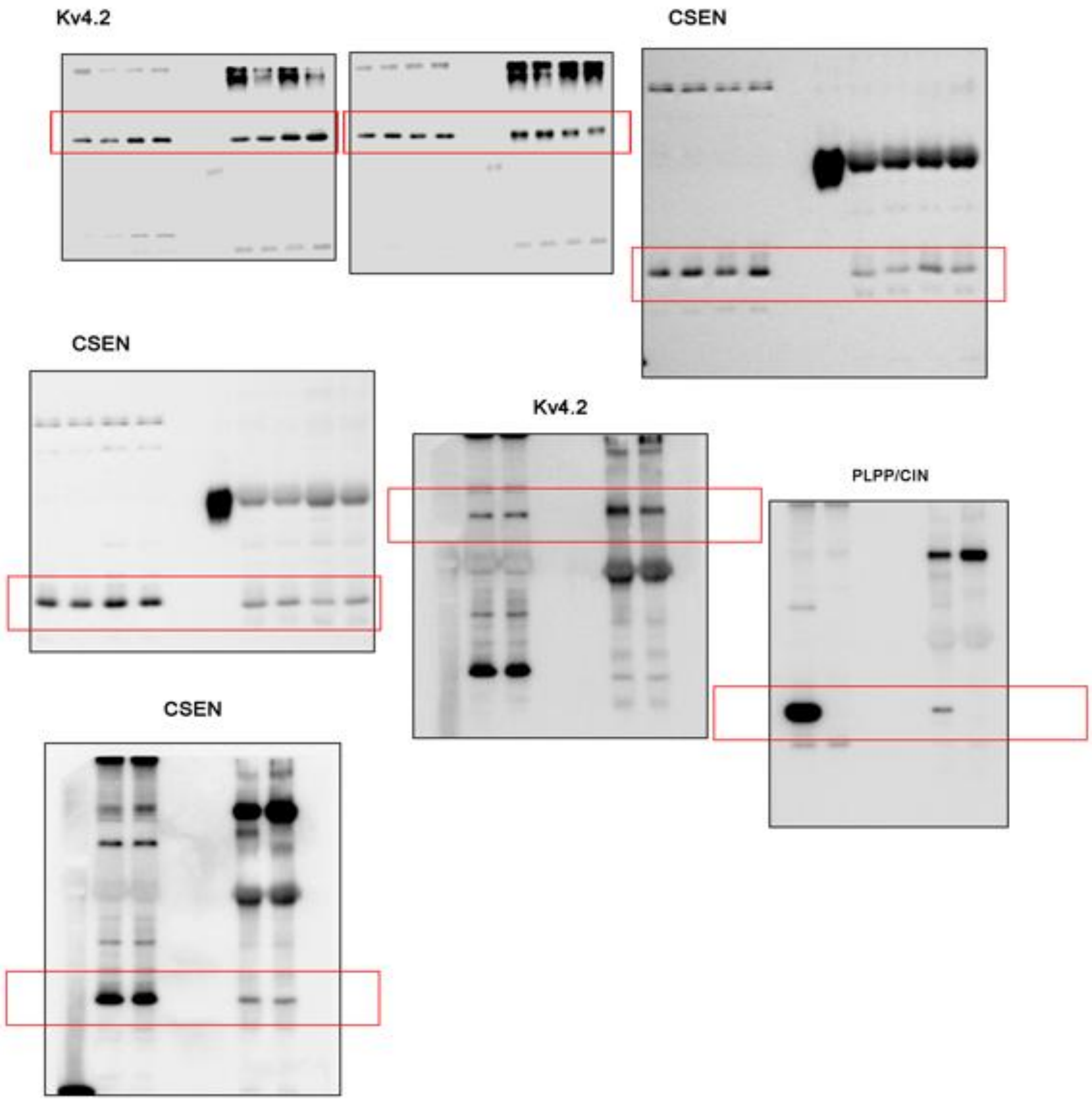

Supplementary Figure 6. : Full-length gel images of western blot data in Fig. 5. The cropped parts of western blots are indicated with boxes.

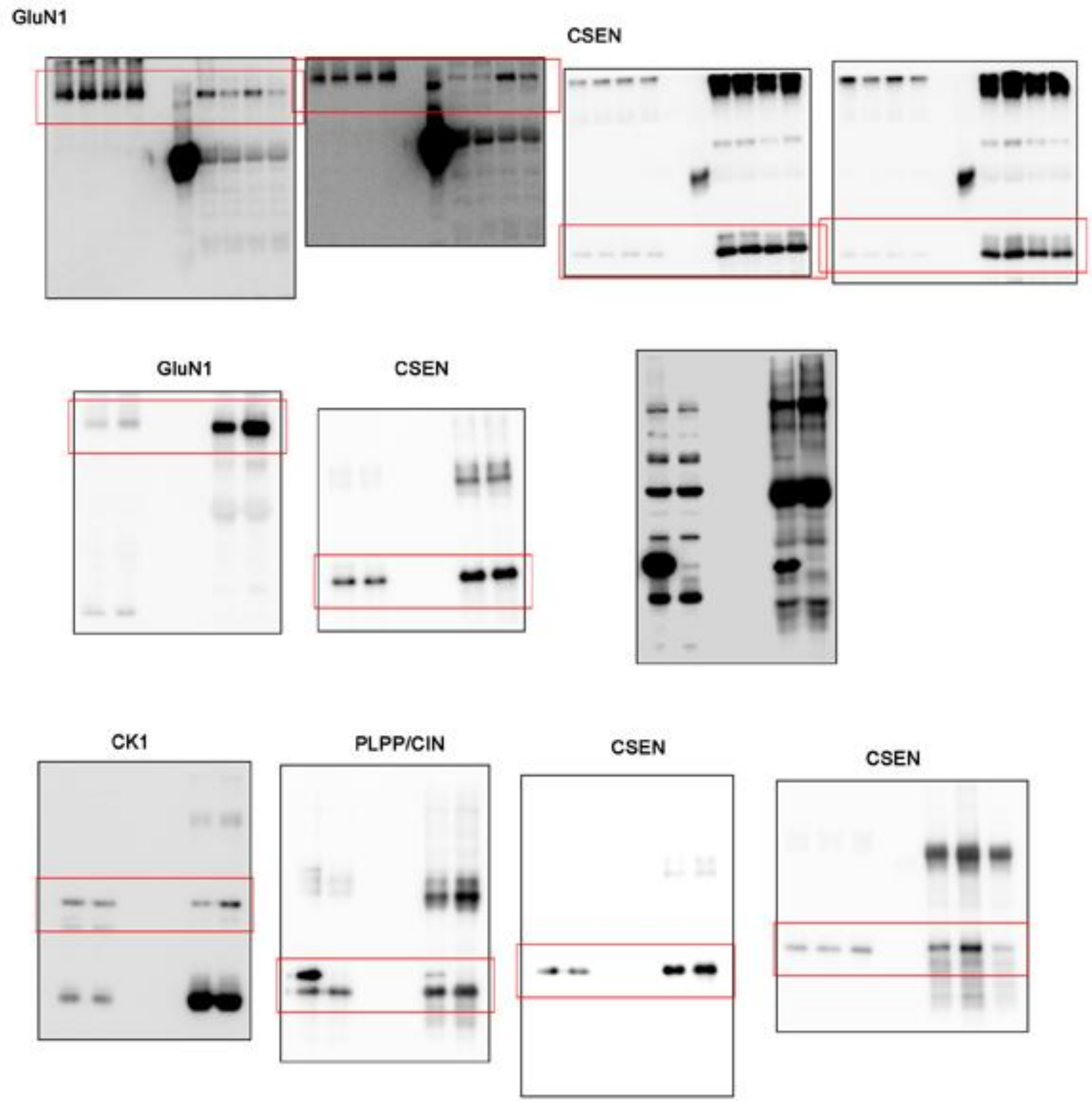

Supplementary Figure 7. : Full-length gel images of western blot data in Fig. 6. The cropped parts of western blots are indicated with boxes.

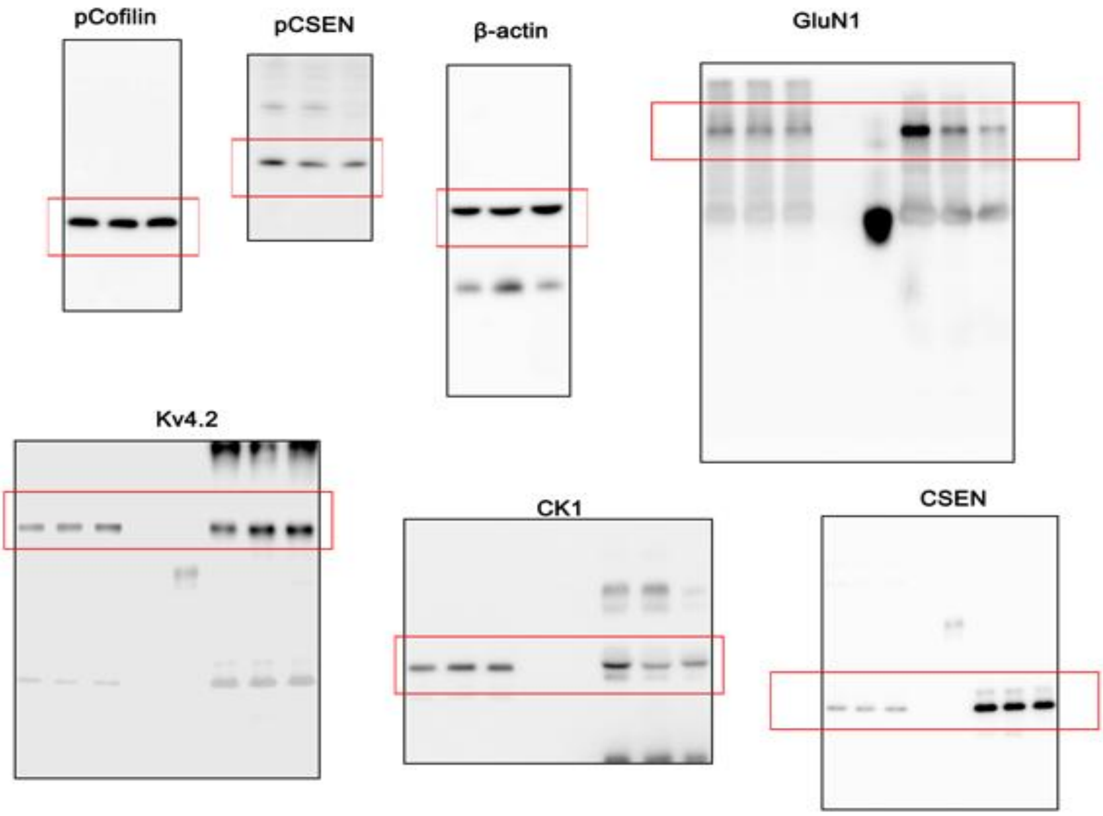

Supplementary Figure 8. : Full-length gel images of western blot data in Fig. 7. The cropped parts of western blots are indicated with boxes.

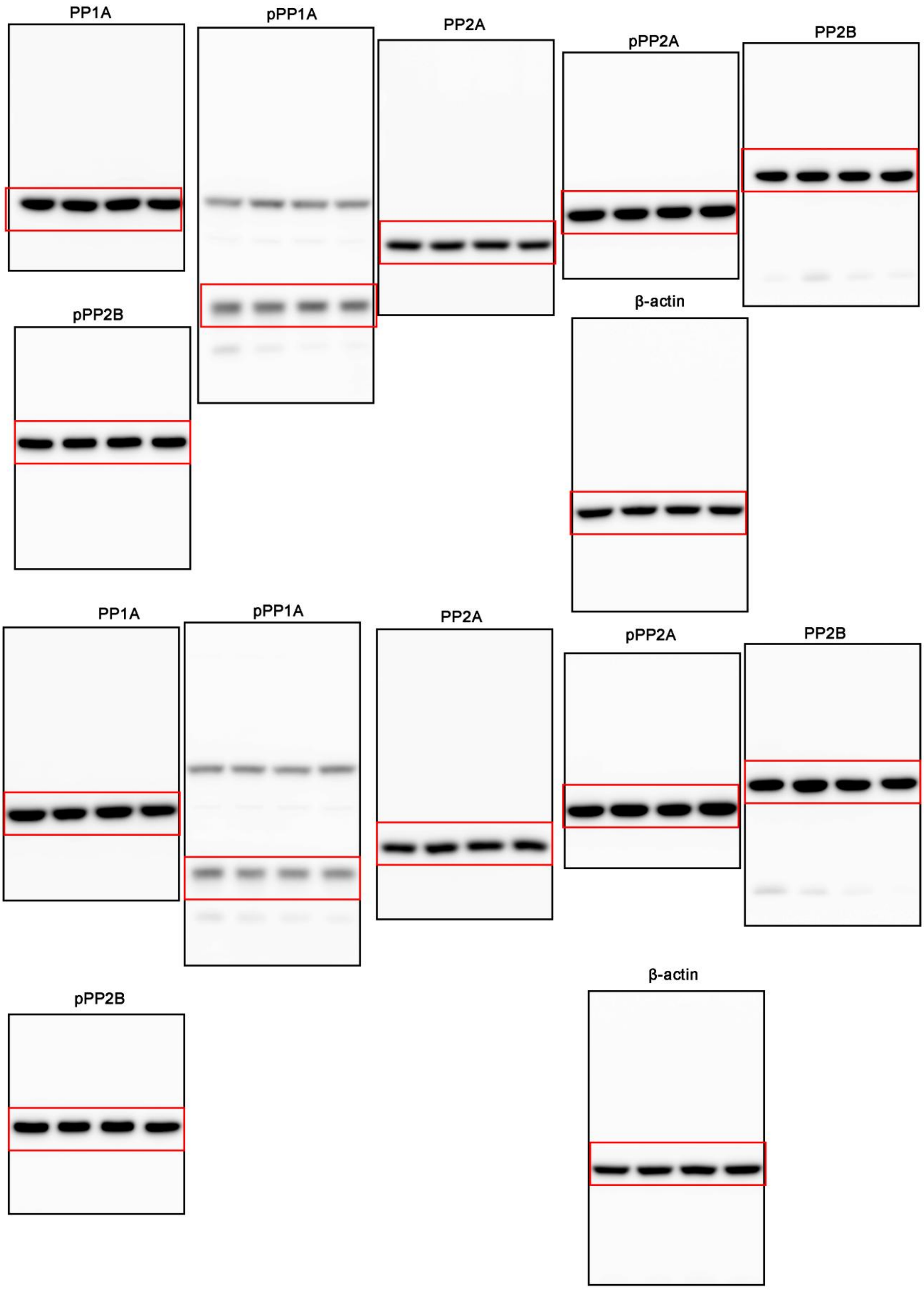

Supplementary Figure 9. : Full-length gel images of western blot data in SFig. 1. The cropped parts of western blots are indicated with boxes.

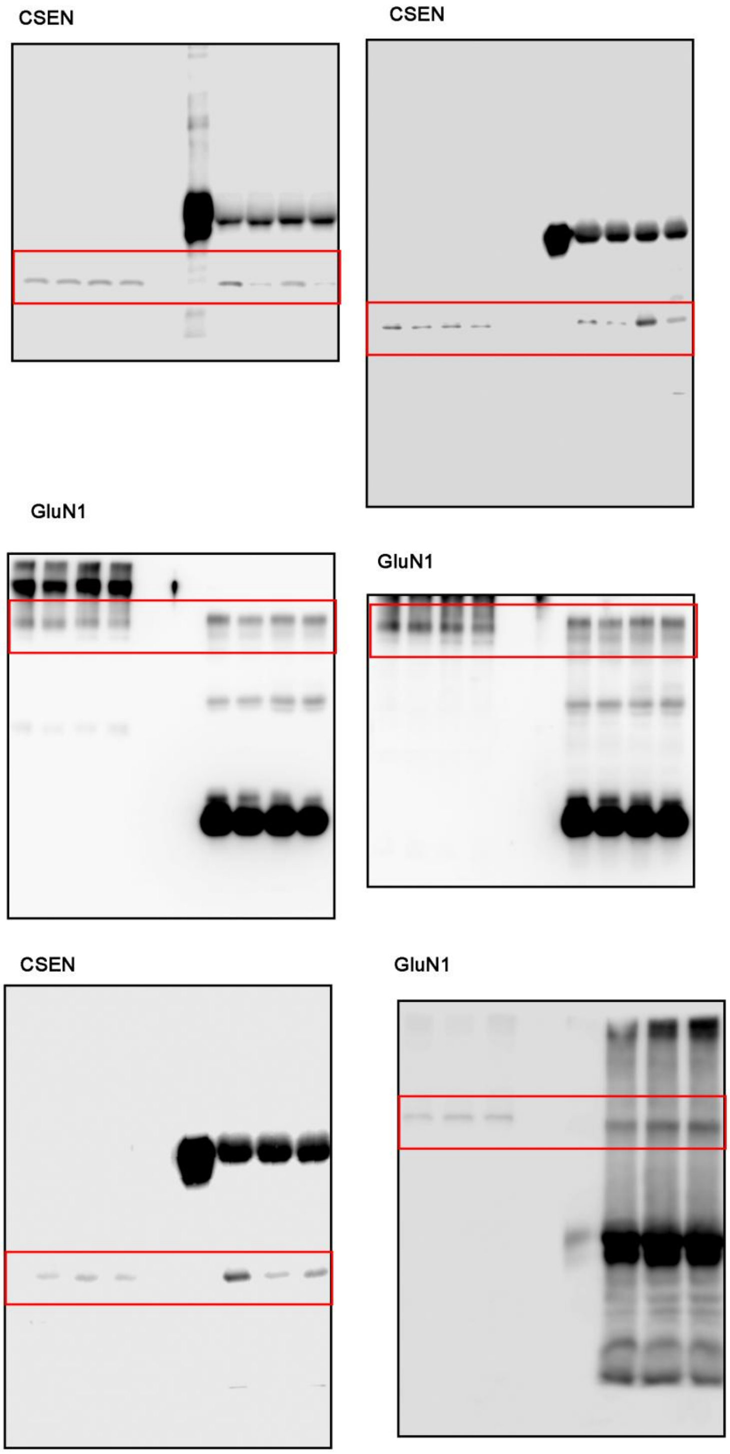

Supplementary Figure 10. : Full-length gel images of western blot data in SFig. 2. The cropped parts of western blots are indicated with boxes.
